# Microplastics in Mussels (*Mytilus galloprovincialis*): Understanding Pollution in Italian Seas

**DOI:** 10.3390/toxics13030144

**Published:** 2025-02-20

**Authors:** Silva Rubini, Martina Munari, Erika Baldini, Filippo Barsi, Daniela Meloni, Nicola Pussini, Francesca Barchiesi, Gabriella Di Francesco, Carmen Losasso, Cristiano Cocumelli, Salvatore Dara, Sebastiano Virgilio, Fabio Di Nocera, Antonio Petrella, Matteo Zinni, Carmela Vaccaro, Negar Eftekhari, Stefano Manfredini, Silvia Vertuani

**Affiliations:** 1 Istituto Zooprofilattico Sperimentale della Lombardia e dell’Emilia Romagna, Ferrara Territorial Office, Via Modena 483, 44124 Ferrara, Italy; silva.rubini@izsler.it (S.R.); martina.munari@izsler.it (M.M.); filippo.barsi@izsler.it (F.B.); 2 Department of Life Sciences and Biotechnology, Faculty of Medicine, Pharmacy and Prevention, University of Ferrara, Via Luigi Borsari 46, 44121 Ferrara, Italy; smanfred@unife.it (S.M.); vrs@unife.it (S.V.); 3 Istituto Zooprofilattico Sperimentale del Piemonte, Liguria e Valle d’Aosta, Via Bologna 148, 10154 Torino, Italy; daniela.meloni@izsplv.it; 4 Istituto Zooprofilattico Sperimentale del Piemonte, Liguria e Valle d’Aosta, Savona—Imperia Territorial Office, Via Martini 6, 17100 Savona, Italy; nicola.pussini@izsplv.it; 5 Istituto Zooprofilattico Sperimentale dell’Umbria e delle Marche, Ancona Territorial Office, Via Cupa di Posatora 3, 60100 Ancona, Italy; f.barchiesi@izsum.it; 6 Istituto Zooprofilattico Sperimentale dell’Abruzzo e del Molise, Via Campo Boario, 64100 Teramo, Italy; g.difrancesco@izs.it; 7 Istituto Zooprofilattico Sperimentale delle Venezie, Viale dell’Università 10, 35020 Legnaro, Italy; closasso@izsvenezie.it; 8 Istituto Zooprofilattico Sperimentale del Lazio e della Toscana, Via Appia Nuova 1411, 00178 Roma, Italy; cristiano.cocumelli@izslt.it; 9 Istituto Zooprofilattico Sperimentale della Sicilia, Via Gino Marinuzzi, 3, 90129 Palermo, Italy; salvatore.dara@izssicilia.it; 10 Istituto Zooprofilattico Sperimentale della Sardegna, Via Duca degli Abruzzi n. 8, 07100 Sassari, Italy; sebastiano.virgilio@izs-sardegna.it; 11 Istituto Zooprofilattico Sperimentale del Mezzogiorno, Via Salute, 2, 80055 Portici, Italy; fabio.dinocera@izsmportici.it; 12 Istituto Zooprofilattico Sperimentale della Puglia e della Basilicata, Via Manfredonia, n. 20, 71121 Foggia, Italy; antonio.petrella@izspb.it; 13 Microbiologia Ambientale e Molecolare—MICAMO Lab, Università degli Studi di Genova, Via Casaregis 50/12, 16129 Genova, Italy; m.zinni@micamo.com; 14 Department of Environmental and Prevention Sciences, University of Ferrara, Via Saragat 1, 44121 Ferrara, Italy; vcr@unife.it (C.V.); ftkngr@unife.it (N.E.)

**Keywords:** microplastics, *Mytilus galloprovincialis*, environmental risk, human health, Raman spectroscopy, marine pollution

## Abstract

Plastic marine litter is a critical issue that threatens marine ecosystems. This study investigated microplastics (MPs) contamination in the Italian seas, involving regions significantly affected by pollution from urban, industrial and agricultural sources. The research, conducted in collaborations between 10 different Experimental Zooprophylactic Institutes throughout Italy, analyzed *Mytilus galloprovincialis* (common mussels) for its filtration capacity and suitability as a bioindicator. Using data from two projects funded by the Italian Ministry of Health, MPs were detected from 7% to 13% of mussel samples, mainly polypropylene and polystyrene fragments and fibers. These findings align with previous studies highlighting the pervasive presence of MPs and their potential risks as mussels are consumed whole, allowing MPs to be ingested. The study underscores the need for standardized detection methods and coordinated policies to mitigate plastic pollution. Public awareness campaigns and improved waste management practices are key to addressing the environmental and health impacts of MPs. Further research on the long-term effects of MPs on marine ecosystems and human health is essential to developing comprehensive mitigation strategies.

## 1. Introduction

The escalating problem of marine litter, particularly the rise in plastic waste, has gained increasing attention due to its extensive impact on marine ecosystems and potential risks to human health [1]. Marine litter, defined as persistent solid material thrown into the marine environment, includes a variety of debris from both land and marine sources. This debris, often carried by rivers, wind, runoff, and urban discharges, ultimately accumulates along coastlines and in the sea [2]. The Adriatic Sea, particularly its northern region, is a critical area for monitoring marine pollution; it is estimated that 40% of marine litter comes from coastal urban populations, another 20% originates from maritime activities, such as shipping and fishing, and the remaining 40% from rivers. A significant input of pollutants comes from the Po River, which serves as a major conduit for urban, industrial and agricultural waste to the sea [3,4]. In a past study on the whole Mediterranean, the western area of the basin was observed to be less contaminated than the eastern one [5]. Also, according to different bibliographies, the Mediterranean Sea seems to be one of the areas most affected by microplastics pollution, with a wide heterogeneity in plastic debris concentrations [1].

Plastics, which make up approximately 80% of the world’s marine litter [6], are characterized by their persistence in the environment. It is estimated that between 1.15 and 2.41 million tons of plastic are dumped into the oceans each year, contributing to the enormous amounts of plastic debris floating in the Pacific and Atlantic Oceans, which cumulatively amount to tens of thousands of tons [7]. Plastics are among the most persistent pollutants and remain in the environment for decades due to their resistance to degradation.

Plastics in the marine environment are categorized by size, ranging from megaplastics over 1000 mm to nanoplastics of less than 1 µm. Among these, microplastics (MPs), defined as particles between 0.1 and 5 mm in size, are increasingly recognized for their widespread presence and their potential to harm marine life and human health [8]. MPs are classified into primary microplastics, which are manufactured to be microscopic for specific industrial and domestic applications, and secondary microplastics, which result from the fragmentation of larger plastic debris through physical, chemical and biological processes. Primary microplastics are intentionally produced for various uses and are included in a variety of cosmetics, including shower gels, facial cleansers, toothpaste, scrubs and powders [9]. MPs are also used in industrial applications, for example, synthetic fibers for clothing, abrasive particles in cleaning products and media used in air blasting [10,11]. Despite campaigns to reduce their use, primary microplastics remain prevalent in the environment and are often released into domestic and industrial wastewater that eventually reaches rivers and seas [12]. Specifically, the most common types of plastics in marine environments include polyethylene (PE), polypropylene (PP), polystyrene (PS), polyethylene terephthalate (PET) and polyvinyl chloride (PVC) [13].

Secondary microplastics, on the other hand, form from the breakdown of larger plastic debris, owing to environmental factors such as UV radiation, temperature and mechanical abrasion [13]. This degradation process is more intense on beaches, where high UV light and physical abrasion by waves accelerate the fragmentation of plastic waste into microplastics [14]. As large plastic objects break down into smaller pieces, their abundance in the marine environment increases, posing a growing threat to marine ecosystems [7,15]. The shape and density of plastic debris influence its distribution within marine environments. Fibers, films and fragments can be found throughout the water column and in benthic sediments, with lighter plastics such as PE and PP typically floating on the surface, whereas denser materials such as PVC, PS and PET settle on the seabed [9]. Over time, aging and degradation processes can alter the properties of plastics, affecting their environmental distribution and ecological impact [16].

Ingestion of MPs by a broad range of marine organisms, from zooplankton to marine mammals, is widely documented, indicating the widespread distribution of these particles in the marine food web [17,18,19]. Studies have shown that ingestion of MPs by these organisms leads to physical damage, such as gastrointestinal blockages and abrasions, and they act as vectors of harmful substances, such as persistent organic pollutants (POPs) and heavy metals [20]. The ingestion of MPs by marine organisms and their potential transfer along the trophic chain raises concerns about their impact on human health, particularly in regions where seafood is a dietary staple [21,22].

Despite numerous studies on the effects of plastics in marine environments, there is a lack of detailed research on specific marine areas where these species are farmed for human consumption. Because of its ecological and economic importance, particularly in the fisheries and aquaculture sectors, the Adriatic Sea is a region where understanding the extent of MPs contamination is crucial for assessing environmental and human health risks.

The present study focuses on the search for MPs in mussels. The commercial Mediterranean mussel (*Mytilus galloprovincialis*), a bivalve that filters the water through its inhalant siphon and retains the small organic and inorganic particles, was chosen as the matrix. Mussels are considered excellent bioindicators and are, therefore, ideal for monitoring environmental contaminants [23].

By evaluating the presence and types of MPs in mussel samples from the Italian sea, two different projects funded by the Italian Ministry of Health were analyzed: the first project is called “Assessment of microplastic contamination in bivalve mollusks reared in Emilia-Romagna (North Adriatic)” (PRC2019015), while the second project is “IIZZSS: the sea on the net” (PRC2021101Strategic). Each project took place over a duration of three years.

By evaluating the presence and types of MPs in mussel samples from the Italian sea, the study proposes to provide a comprehensive assessment of contamination levels and develop strategies to mitigate their impact. The presence of macroplastics and microplastics in marine environments is a global issue that requires urgent attention. Understanding the extent of contamination and its ecological and health implications is essential to developing effective strategies to combat this growing problem.

## 2. Materials and Methods

### 2.1. Study Area and Sampling

#### 2.1.1. Assessment of Microplastic Contamination in Bivalve Mollusks Reared in Emilia-Romagna (North Adriatic)—PRC2019015PRC2019015

This study was conducted in the northern Adriatic Sea, focusing on six geo-referenced farming areas classified as “zone A”, designated for mussels’ cultivation for direct human consumption. These areas are located 3 to 6 miles south of the Po River mouth, in Emilia-Romagna, a region known for its significant pollution due to urban, industrial and agricultural runoff [4,24]. Mussel samples were collected as part of the Mollusk Bivalve Surveillance Plan, with samples delivered to the Zooprophylactic Institute of Lombardy and Emilia-Romagna (Ferrara section) for analysis. The samples were collected and analyzed every month from 2020 to June 2023, i.e., about 5 samples per month for a total of 146 samples. The last 6 months were dedicated to completing the analysis and drawing up the final report.

#### 2.1.2. IIZZSS: The Sea on the Net—PRC2021101Strategic

**Figure 1:**
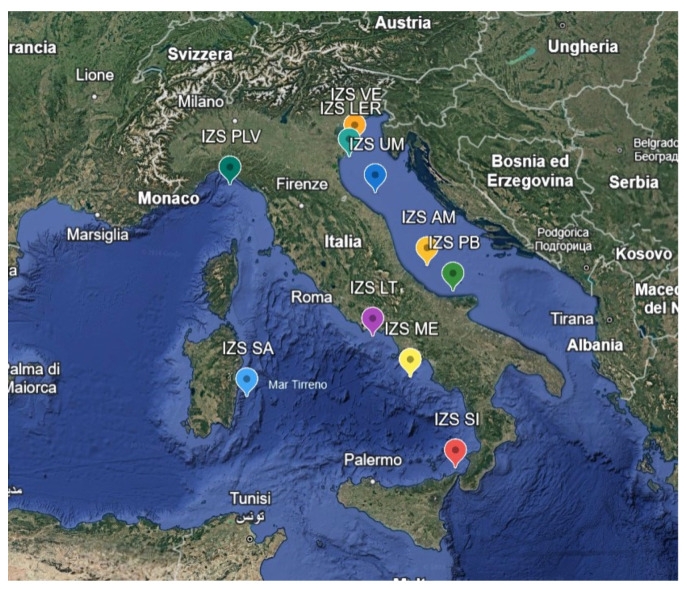
Details of the various sampling areas of the PRC2021101Strategic project.

This research was conducted in the Italian Sea, thanks to the cooperation of the Zooprofilatic Institutes involved in the project. Mussel samples were collected as part of the Mollusk Bivalve Surveillance Plan, with samples delivered to the Zooprophylactic Institute of each region for analysis (Table 1, Figure 1). The samples were collected and analyzed throughout the year for the whole duration of the project, from 2021 to June 2024. The Ferrara laboratory received a total of about 10 samples per IZS from each Zooprophylactic Institute for a total of 104 samples.

### 2.2. Sample Collection and Preservation

Due to the COVID-19 pandemic, there were delays in processing the samples, and the collected mussels were frozen at −20 °C to preserve them until analysis. Once thawed, the mussels were dissected, and their soft tissues were pooled for each sampling site to create composite samples, ensuring a representative analysis of each area.

### 2.3. Contamination Prevention Measures

To prevent contamination during the analytical process, all procedures were conducted under strict contamination controls. Laboratory personnel wore cotton-fiber gowns and gloves, and all instruments used were made of glass or metal. Additionally, all water used in the analysis was deionized and microfiltered to minimize the risk of introducing extraneous MPs into the samples [10].

### 2.4. Sample Digestion and MP Extraction

Mussel soft tissues were subjected to chemical digestion using a 10% potassium hydroxide (KOH) solution [25]. In fact, with KOH, organic matter can be effectively digested without damaging most of the plastic polymers analyzed. The protocols were carried out under a fume hood, again to limit contamination of the samples. At the end of digestion, glass fiber filters placed on a vacuum pump were used to filter the solution. For each sample, 100 g of pulp and intravalvular liquid were homogenized with a rotary homogenizer. Subsequently, 1 g was taken from each sample, placed inside a glass flask and combined with a volume of 10% KOH equal to 3 times the weight of the homogenate. To avoid external contamination, the flask was closed with aluminum foil and incubated at 60 °C for 48 h. At the end of the incubation time, the solution in the Erlenmeyer flask was filtered through a vacuum pump onto fiberglass filters with a pore size of 0.1 mm to capture potential MPs. After filtration, the filters were rinsed with deionized water to remove any remaining chemicals, ensuring that only MPs remained for analysis. The filters were then placed in glass Petri dishes and dried in an oven at 37 °C until dry.

### 2.5. Microscopic and Spectroscopic Analysis

The filtered materials were initially examined with the Olympus SZ60^®^ stereomicroscope(New York Microscope Company, Hicksville, NY, USA), using magnifications from 20 to 60x, to identify potential MPs based on their physical characteristics, such as size, shape and uniform color. Suspected MPs were subsequently analyzed using Raman spectroscopy, which provides precise identification of the chemical composition of the particles by analyzing their vibrational spectra [26]. Raman spectroscopy is particularly useful in distinguishing different types of polymers, including polypropylene, polystyrene and polyvinyl chloride, which are commonly found in marine environments [27,28].

### 2.6. μ-Raman Spectroscopy

μ-Raman spectroscopy was used to verify the identity of the fluorescing plastic particles found on the filters. The μ-Raman spectroscopy was carried out with a LabRam HR800 spectrometer (Horiba Scientific, Kyoto, Japan) with a focal length of 800 mm, equipped with a digital camera and coupled with an Olympus BXFM optical microscope(Olympus, Grenchen, Switzerland). The spectrometer was equipped with an air-cooled CCD detector (1024 × 256 pixels) set at −70 °C, and, with 600 grooves/mm grating and a 10x, 50x and 100x objective, was used to send the collected signals to the detector. The excitation source was a He-Ne laser (632.8 nm line) with a maximum laser power of 17 mW, controlled over the sample using ND filters(The Tiffen Company, Hauppauge, NY, USA). The Raman spectra were recorded in the range of 100–4000 cm^−1^ with an exposure time varying between 3 and 20 s and 3–15 accumulations. The spectrometer was calibrated with silicon at 520 cm^−1^.

## 3. Results

### 3.1. Microplastic Detection and Characterization

In this study, samples from two projects funded by the Italian Ministry of Health were analyzed: the first project was called “Assessment of microplastic contamination in bivalve mollusks reared in Emilia-Romagna (North Adriatic)” (PRC2019015), while the second project was “IIZZSS: the sea on the net” (PRC2021101Strategic). Each project had a duration of three years.

**Figure 2:**
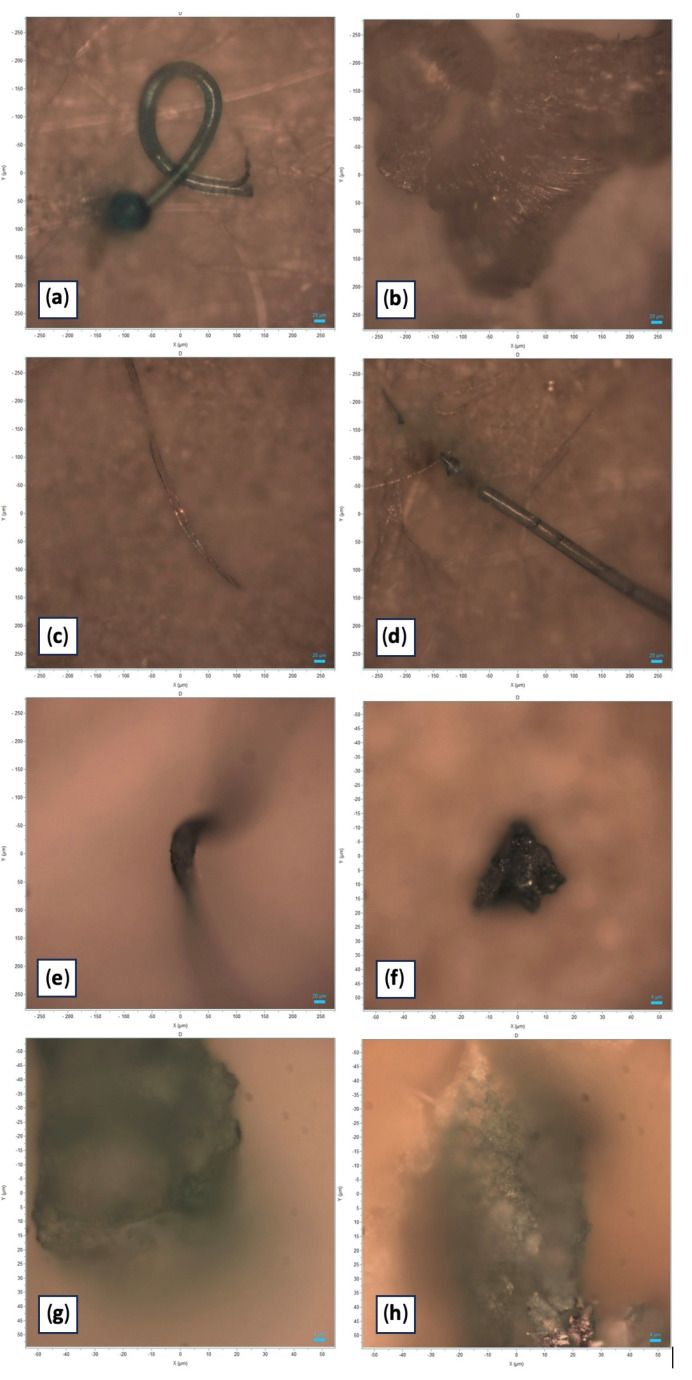
Photographic documentation of the microplastic particles analyzed (original images obtained with Horiba Scientific’s LabSpec 6). (**a**) Light blue polystyrene filament characterized by “Pygmosol green” dye; size: 580 µm. (**b**) Transparent polystyrene filament; size: 581 µm. (**c**) Blue PVC filament characterized by “Egypthian Blue” dye; size: 456 µm. (**d**) Light blue filament characterized by “Hostasol green” dye; size: 393 µm. (**e**) Green polypropylene fragment characterized by “Pigmosol green” dye; size: 171 µm. (**f**) Green polystyrene fragment characterized by “Copper phthalocyanine” dye; size: 32 µm. (**g**) Green fragment of polystyrene characterized by “Irgalith blue” dye; size: 95 µm. (**h**) Blue-green fragment characterized by “Cobalt phthalocyanine” dye; size: 114 µm.

**Figure 3:**
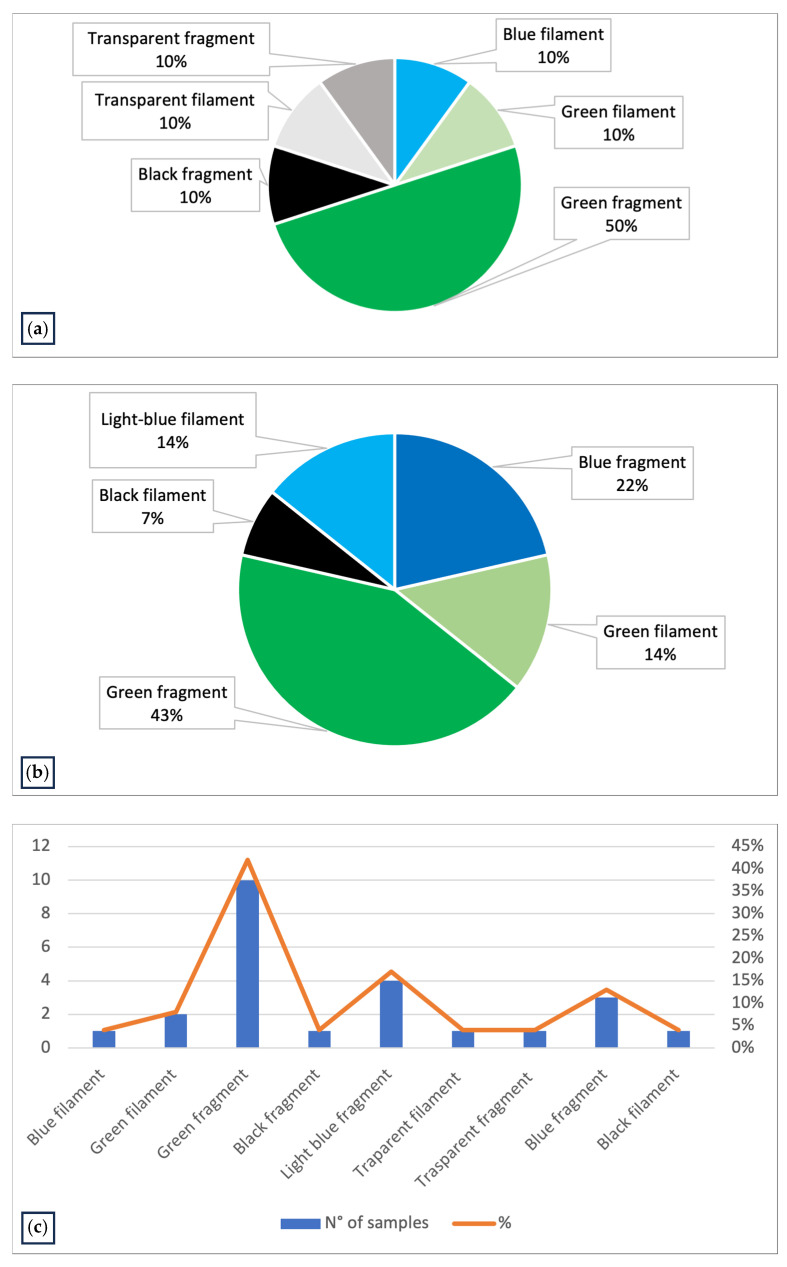
(**a**) Graph representing the characteristics of MPs identified in the *Mytilus galloprovincialis* samples collected and analyzed in Emilia-Romagna region in PRC2019015; (**b**) graph representing the characteristics of MPs identified in the *Mytilus galloprovincialis* samples of PRC2020101Strategic, collected in Italian coasts and analyzed in Ferrara laboratory; (**c**) graph representing the characteristics of MPs identified in all samples analyzed (PRC2019015 and PRC2020101Strategic).

The first study, PRC2019015, was conducted from 2020 to 2023 in the Emilia-Romagna region. A total of 146 samples were collected from different farming sites and mussel samples were analyzed, revealing MPs in 7% of the samples. Upon visual examination under the stereomicroscope, each filter had 1 to 5 particles identified as potential MPs based on their physical characteristics: maximum diameter less than 5 mm, uniform color, uniform thickness along the entire length (if fibers) and absence of organic debris adhering to the surface. The MPs identified were mainly fragments (70%) and fibers (30%), ranging in size from 32 to 581 µm (Figure 2). The colors of the MPs varied, the most common being blue (30%), green (60%) and transparent (10%) particles (Figure 3a).

**Figure 4:**
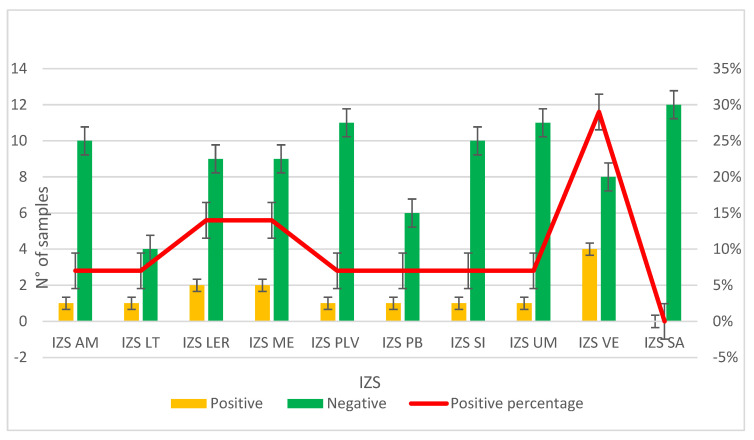
Graph representing the results on the presence or absence of MPs in samples provided by the Experimental Zooprophylactic Institutes (IZS) involved in the PRC2020101Strategic project.

The second study, PRC2021101Strategic, was conducted from 2021 to 2024. Samples were collected from different farming sites in various regions of Italy; specifically, 10 different Experimental Zooprophylactic Institutes (IZS) throughout Italy delivered their mussels to Ferrara Laboratory for MPs analysis. A total of 104 mussel samples were analyzed, revealing MPs in 13% of the samples (Figure 4). Upon visual examination under the stereomicroscope, each filter had one to five particles identified as potential MPs based on their physical characteristics: maximum diameter less than 5 mm, uniform color, uniform thickness along the entire length (if fibers) and absence of organic debris adhering to the surface. The MPs identified were fragments (64%) and fibers (36%). The colors of the MPs varied, the most common being green (57%) and blue (36%) particles (Figure 3b). The characteristics of the MPs highlighted in Figure 3c represent the totality of the data regarding color and type of plastic identified in both projects (Table 2).

### 3.2. Samples Composition

#### 3.2.1. Types of Plastic Polymer Identified

**Figure 5:**
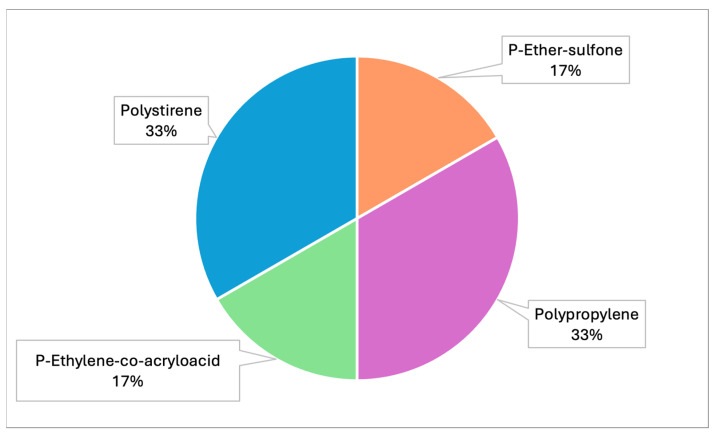
Graph representing the different types of plastic polymers identified by Raman (PRC2019015 and PRC2020101Strategic).

**Figure 6:**
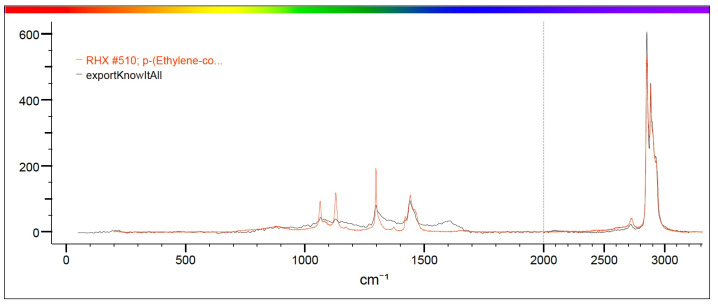
The figure shows in red the characteristic spectrum of P-Ethylene-co-acryloacid present in the library and in black the spectrum of the analyzed sample. The correspondence between the two spectra is such that it can be stated that the sample identified in the mussel is P-Ethylene-co-acryloacid.

**Figure 7:**
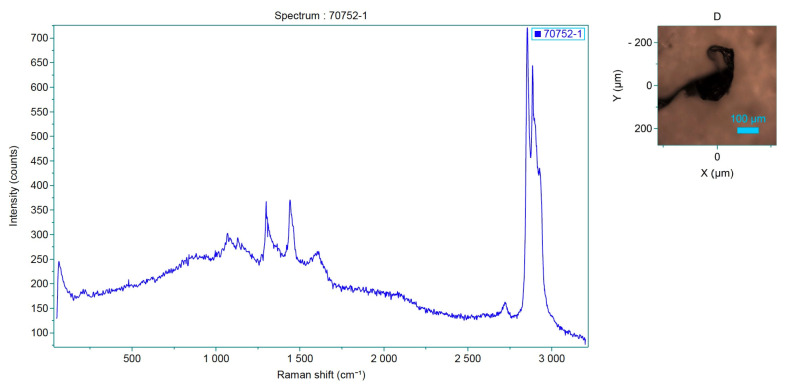
Spectrum of the analyzed sample, which corresponds to P-Ethylene-co-acryloacid.

Raman spectroscopy identified polypropylene, polystyrene and polyvinyl chloride (PVC) as the main polymers found in MPs. In detail, samples from both projects were analyzed by Raman and the spectra obtained were compared with those available in the library. Specifically, correspondence with the following plastic polymers was confirmed in six samples analyzed (Table 3, Figure 5): P-Ether-sulfone, polypropylene, P-Ethylene-co-acryloacid (Figure 6 and Figure 7) and polystyrene.

These materials are widely used in various industries and are known to be persistent in the marine environment due to their resistance to degradation.

#### 3.2.2. Types of Dyes Identified

**Figure 8:**
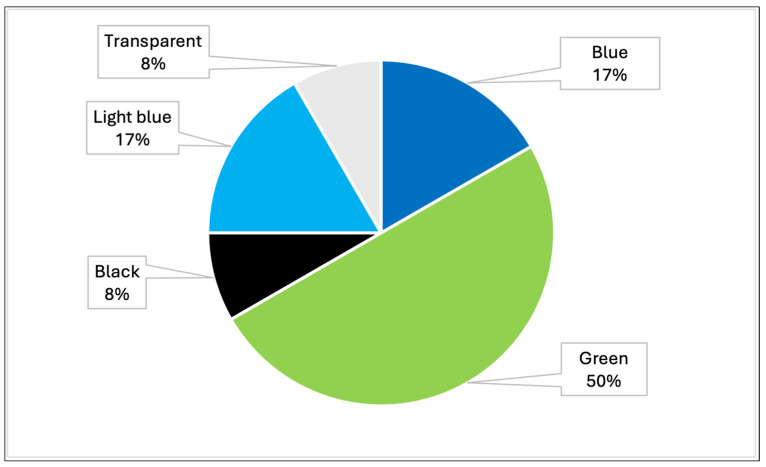
Graph representing the percentages of the different colors detected in MPs in all samples analyzed throughout Italy (PRC2019015 + PRC2020101Strategic).

**Figure 9:**
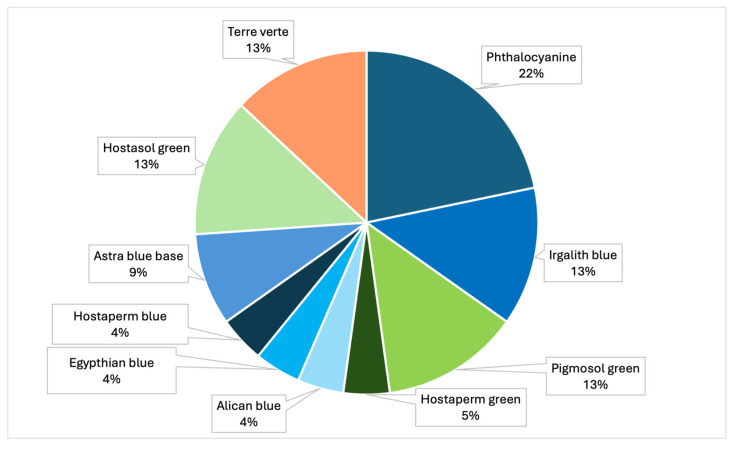
Graph representing the percentages of the different types of dyes identified by Raman (PRC2019015+ PRC2020101Strategic).

**Figure 10:**
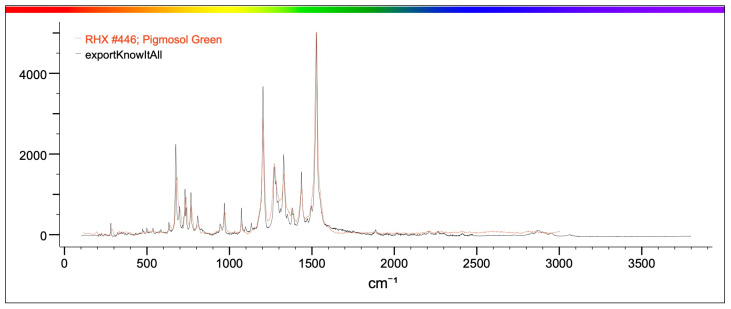
The figure shows in red the characteristic spectrum of Pigmosol green dye present in the library and in black the spectrum of the analyzed sample. The correspondence between the two spectra is such that it can be stated that the sample identified in the mussel is Pigmosol green.

**Figure 11:**
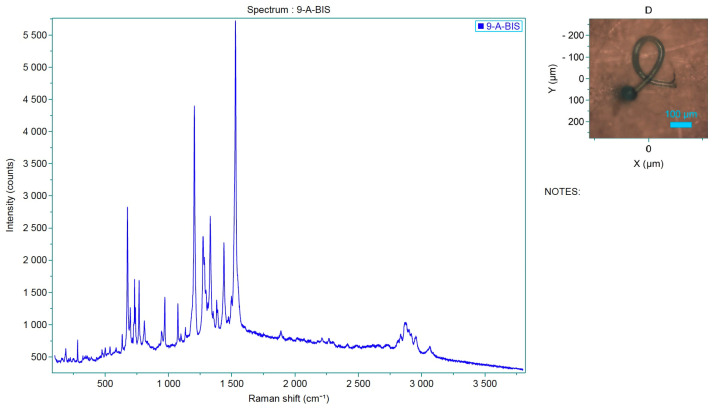
Spectrum of the analyzed sample, which corresponds to Pigmosol green dye.

The samples analyzed showed that the most prevalent colors were, mainly, green, blue, light blue, black and transparent (Table 4, Figure 8). In particular, analysis by Raman spectroscopy identified a number of dyes in 23 samples, which are shown in Table 5 and Figure 9. The dyes in question that have been reported as characterizing plastic polymers are: phthalocyanine, Irgalith blue, Pigmosol green (Figure 10 and Figure 11), Hostaperm green, Alican blue, Egypthian blue, Hostaperm blue, Astra blue base, Hostasol green and Terre verte.

### 3.3. Spatial Distribution of MPs

MP pollution comes from human activity. The spatial distribution of MP contamination can be varied across the sites. This variation may be attributed to differences in local pollution sources, such as proximity to urban or industrial areas, and differences in hydrodynamic conditions that influence the distribution and accumulation of MPs in the water column [21,29].

The most contaminated regions are Veneto and Emilia-Romagna, located in the northern Adriatic Sea. The Po River flows through northern Italy and throughout the Po Valley, an area characterized by high population density and industrialization; it flows into the Adriatic Sea, carrying pollution from industrial waste, livestock farms and urban waste.

Campania is the third region with the highest positive numbers. Here, although not all possible causes are known, it is speculated that the high values found are due to the proximity of farms to inhabited areas where population density is high.

In the region of Sardinia, on the other hand, no positive events occurred, indicating that high currents favor higher recurrence, although the region is characterized by heavy traffic and affected by high tourism.

## 4. Discussion

### 4.1. Environmental and Human Health Implications

The detection of MPs in *Mytilus galloprovincialis* from the northern Adriatic Sea and Italian coast underscores the potential risks associated with the consumption of contaminated seafood. Mussels are typically consumed whole, including their digestive tracts, providing a direct pathway for MPs to enter the human body. The bioaccumulation of MPs in marine organisms, combined with their ability to transport harmful substances, such as persistent organic pollutants (POPs) and heavy metals, poses a significant threat to human health [20,30,31]. Ingestion of MPs by marine organisms and their potential transfer along the trophic chain raises concerns about long-term impacts on marine biodiversity and ecosystem health [15,32]. 

The positivity of 7% of mussels in the first study and 13% in the second highlights how the presence of MPs in edible matrices can compromise food safety and human health. Due to their small size, MPs can be absorbed and potentially distributed to different organs through the circulatory system [33]. Studies have shown that microplastics can persist in the human gut longer than other ingested substances, even though most of the plastic is excreted after ingestion. The presence of MPs in different ecosystems affects the food web through the trophic transfer of microplastics themselves along the food chain. However, some evidence suggests that MPs may translocate from the digestive tract into the bloodstream, raising concerns about potential accumulation in critical organs such as the liver and kidneys [23,34,35]. Furthermore, MPs can act as vectors for toxic chemicals, including endocrine-disrupting compounds, which have been linked to reproductive and developmental abnormalities in both wildlife and humans [30,36].

Previous studies, especially on marine species, have demonstrated that microplastic particles can be ingested at different trophic levels and through different types of feeders (detritivores, filter feeders, and predators). The ingestion of microplastic particles (MPs) by marine invertebrates has demonstrated several health effects on animals, including reduced reproduction, impaired growth of individuals, and decreased overall fitness [33,37]. Additionally, MPs can cause internal damage such as lacerations, inflammatory reactions, and feelings of false satiety, leading to reduced nutrition and decreased nutrient intake and energy [33] depletion [35,38]. 

Plastics often contain chemical additives that have physical functional properties such as elasticity, stiffness, UV stability, flame retardancy, and depigmentation. Furthermore, MPs can adsorb heavy metals, environmental contaminants, and persistent organic pollutants, and pathogenic bacteria can concentrate on the surface of plastic materials from the surrounding marine environment, increasing the risk of human exposure to seafood consumption [20,39,40]. Microbial adhesion and growth on the surface of microplastic synthetic polymers can interact with the bacterial flora of the intestinal tract [34,35]. It can also alter gut microbiota and potentially trigger immune responses in both marine organisms and humans [39]. However, the potential long-term health effects on human health from exposure to MPs remain poorly understood, and this information, along with the results obtained from studies of population dietary habits, plays a key role in helping to assess consumer risk, necessitating further research into their potential for systemic toxicity and bioaccumulation [35,41].

### 4.2. Comparison with Other Studies

The results of this study are consistent with previous research conducted in the Adriatic Sea, other regions of Italy with high human activity [7,9], and other marine environments, where MPs have been detected in various marine species, including fish, crustaceans, and mollusks [17,23,34,36,37]. The types of polymers identified in this study (polypropylene, polystyrene, and polyvinyl chloride) are among the most commonly found types of plastics in the marine environment, being the most widely used plastics worldwide, reflecting their widespread industrial use and persistence in aquatic ecosystems [13,33,38].

MP contamination in marine organisms is not only a human health concern but also an ecological issue. MP ingestion by marine species has been shown to cause physiological stress, reduced filtration efficiency, and immune responses in filter-feeding organisms such as mussels. Additionally, the trophic transfer of MPs through the food web raises concerns about bioaccumulation in higher predators, including commercially important fish species [15,32,42].

Similar contamination levels have been reported in other Mediterranean regions, emphasizing the widespread nature of MP pollution [1]. This highlights the necessity for comprehensive monitoring and risk assessment strategies to mitigate MPs’ impact on both food safety and marine ecosystems [36].

### 4.3. Methodological Considerations

The extraction method used to digest the tissue samples proved effective and could, therefore, be used to serve as a standardized method to study the presence of microplastics in various biological matrices. The use of Raman spectroscopy in this study provided accurate polymer identification of MPs, although some challenges were encountered, such as fluorescence interference, which may have been caused by the presence of biofilm attached to the particle or the presence of pigments and additives that prevent the acquisition of a clean spectrum [12]. These challenges underscore the need for further refinement of analytical techniques to improve the accuracy and reliability of MP identification in complex biological matrices. In any case, Raman spectroscopy is an efficient method of chemical analysis, which also provides information about the crystal structure of polymer samples [12].

Color is one of the crucial characteristics of plastics when using visual identification. The morphological description of microplastics is based on MPs and considers aspects such as origin, type, shape, color, and/or degree of degradation level. In the case of many biological residues some cases, colored plastics can facilitate the identification process. Color is a tool to assess the level of and can provide insights into photodegradation, the residence time of a particle on the water surface, and the degree of fouling and erosion [12]. Future research should prioritize the development of non-destructive and high-throughput detection methods to enhance large-scale environmental monitoring and risk assessment.

### 4.4. Implications for Future Research and Policy

The results and findings of this study underscore the importance of the ongoing need for continuous monitoring and research to better understand the extent of MP contamination in marine environments and its implications for human health. The consistent detection of MPs across multiple studies suggests that plastic pollution is a pervasive issue that requires coordinated efforts at both the national and international levels. Future research should focus on refining analytical methods to overcome challenges such as fluorescence interference in Raman spectroscopy and exploring the long-term effects of MP exposure on marine organisms and humans [22,39,40]. In addition, there is an urgent need to develop standardized methodologies for MP detection and characterization to facilitate comparisons across different studies and regions, which is critical for developing effective mitigation strategies [8]. This is a persistent issue that requires coordinated efforts at both national and international levels [22,35,41].

Future research should prioritize:

- Refining analytical methods: Addressing challenges such as fluorescence interference in Raman spectroscopy and improving polymer identification techniques.
- Standardizing detection protocols: Establishing internationally recognized methodologies for MP characterization to enable cross-study comparisons [8].
- Assessing long-term toxicity: Conducting studies to determine the chronic effects of MP exposure on marine organisms and humans, particularly concerning bioaccumulation and systemic toxicity [33,41].
- Evaluating ecological impact: Investigating the effects of MP ingestion on marine biodiversity, trophic transfer, and food web stability [15,32,42].

The findings also support the call for stronger regulatory measures to reduce plastic pollution at the source. Policies aimed at reducing the production and use of single-use plastics, improving waste management practices infrastructure, and promoting the development of biodegradable alternatives are essential to mitigate the critical steps toward mitigating MPs’ impact of MPs on marine ecosystems and public health. Additionally, public awareness about the environmental and health risks associated with MP campaigns is crucial for driving and fostering behavioral changes that can contribute to reducing plastic waste and encouraging sustainable consumption patterns.

## 5. Conclusions

This study confirms the significant presence of microplastics across the Italian seas, with *Mytilus galloprovincialis* serving as an effective bioindicator for environmental contamination. The detection of MPs in 7% to 13% of samples highlights the pervasive nature of plastic pollution and its potential risks to ecosystems and human health. These findings call for the implementation of comprehensive and harmonized monitoring programs to assess the distribution of MPs more consistently, including their pathways and seasonal variations. Addressing this problem requires a multifaceted approach. Firstly, policymakers must strengthen regulatory frameworks to reduce plastic emissions at their sources, including single-use plastics, industrial discharges and inefficient waste management systems. Secondly, technological advancements are needed to improve MPs detection methodologies and facilitate large-scale monitoring. Moreover, awareness campaigns targeting both industries and the public to promote responsible consumption, recycling and plastic alternatives are essential. Future research should focus on understanding the long-term effects of MP ingestion, particularly with regard to mechanisms of bioaccumulation within different trophic levels, their persistence in human tissues and the potential impacts on cellular and systemic functions on human health and marine organisms, particularly bioaccumulation in the food chain. Comparative studies across multiple geographic regions will provide a broader perspective on trends in plastic pollution and its causes. Finally, interdisciplinary collaboration is essential to design and implement mitigation strategies that address the environmental and socio-economic dimensions of plastic pollution. By fostering innovation and international cooperation, significant progress can be made toward the safeguarding of marine ecosystems and the sustainable use of ocean resources.

## Figures and Tables

**Table 1:** Zooprophylactic institutes (IZS) involved in the project PRC2020101Strategic and their coordinates, which correspond to the sampling areas.

Zooprophylactic Institute (IZS)	Region	Coordinates
IZS PLV	Liguria	44.07805—9.86388
IZS UM	Marche	43.75473—13.2238
IZS AM	Abruzzo	42.31802—14.49231
IZS VE	Veneto	45.33744—12.30144
IZS LT	Lazio	41.0151—13.56495
IZS SI	Sicilia	38.26722—15.63444
IZS LER	Emilia-Romagna	44.74333—12.34337
IZS SA	Sardegna	39.94667—9.67939
IZS ME	Campania	40.78521—14.35562
IZS PB	Puglia	41.92558—15.29414

**Table 2:** Characteristics of MPs identified in all samples analyzed (PRC2019015 and PRC2020101Strategic).

MPs Identified	N° of Positive Samples	%
Blue filament	1	4%
Green filament	2	8%
Green fragment	10	42%
Black fragment	1	4%
Light blue fragment	4	17%
Transparent filament	1	4%
Transparent fragment	1	4%
Blue fragment	3	13%
Black filament	1	4%

**Table 3:** Percentages of different plastic polymers detected by Raman in all samples analyzed throughout Italy (PRC2019015+ PRC2020101Strategic).

Type of Polymers	N° of Samples	%
P-Ether-sulfone	1	17%
Polypropylene	2	33%
P-Ethylene-co-acryloacid	1	17%
Polystirene	2	33%

**Table 4:** Percentages of the different colors detected in MPs in positive samples analyzed throughout Italy (PRC2019015 + PRC2020101Strategic).

Color of Plastic	N° of Positive Samples	%
Blue	4	17%
Green	12	50%
Black	2	8%
Light blue	4	17%
Transparent	2	8%

**Table 5:** Table representing the types of dyes identified in positive samples analyzed (PRC2019015 and PRC2020101Strategic).

Type of Dyes	N° of Positive Samples	%
Phthalocyanine	5	21.74%
Irgalith blue	3	13.04%
Pigmosol green	3	13.04%
Hostaperm green	1	4.35%
Alican blue	1	4.35%
Egypthian blue	1	4.35%
Hostaperm blue	1	4.35%
Astra blue base	2	8.7%
Hostasol green	3	13.04%
Terre verte	3	13.04%

## Data Availability

The raw data supporting the conclusions of this article will be made available by the authors on request.
